# Climate‐Induced Changes in Habitat Suitability for a Cold‐Water Endemic Species in the Lancang River Basin: A Case Study of *Schizothorax lantsangensis*

**DOI:** 10.1002/ece3.72753

**Published:** 2025-12-22

**Authors:** Yuchen Xu, Jia Li, Li Chang, Ruidong An

**Affiliations:** ^1^ State Key Laboratory of Hydraulics and Mountain River Engineering Sichuan University College of Water Resources and Hydropower Chengdu PR China; ^2^ Guiyang Engineering Co., Ltd. Power China Guiyang PR China

**Keywords:** climate change, habitat suitability, MaxEnt, river flow, *Schizothorax lantsangensis*, water temperature

## Abstract

Climate change is reshaping plateau freshwater ecosystems, where interacting thermal, hydrological, and topographic gradients structure fish distributions. Understanding how biological responses align with high‐altitude freshwater conditions is therefore essential for anticipating the future of endemic fishes and informing sustainable management in the Lancang River Basin (LRB). *Schizothorax lantsangensis* , an endemic cold‐water species of socio‐economic value in the upper–middle LRB, was modeled using MaxEnt with river flow, seasonal water temperatures (typical wet/dry years), topography, and bioclimatic variables; projections were generated under four Shared Socioeconomic Pathways (SSPs) for 2061–2080 and 2081–2100. River flow accumulation, winter (dry‐year) water temperature, elevation, and spring (dry‐year) water temperature emerged as the dominant drivers of suitability. At present, 4776.67 km of river length (22.1% of the network) is suitable, concentrated in the upper and middle basin near Nangqen (NQ), Qamdo (QD), Chagyab (CY), and Dêqên (DQ). Response curves identify an occupancy window defined by flow accumulation of 855–14,835, winter 3.5°C–6.0°C, spring 8.5°C–11.0°C, and elevations of 2722–3650 m. Future warming drives consistent contraction across scenarios, with losses reaching up to 30.63% by the 2090s under SSP585. These quantified benchmarks provide a direct basis for conservation and management by (i) prioritizing reserve designation and connectivity in the identified upper–middle reaches, (ii) safeguarding cold‐water refugia and high‐accumulation corridors, and (iii) guiding seasonal environmental‐flow and thermal‐mitigation actions to keep spring temperatures near 8.5°C–11.0°C (winter 3.5°C–6.0°C), using the current 4776.67 km (22.1%) extent as a monitoring and planning baseline.

## Graphical abstract

This study evaluates the ecological suitability of *S. lantsangensis* using environmental variables including river flow, water temperature, topography, and bioclimatic factors.

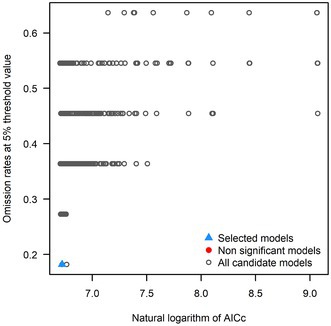

## 1 Introduction

Freshwater ecosystems cover a small share of the Earth's surface but support a large fraction of global biodiversity and essential ecosystem services. They have declined under combined pressures that include overexploitation, pollution, flow alteration, habitat degradation, and biological invasions (Dudgeon et al. 2006; Reid et al. 2019; Tickner et al. 2020). Recent syntheses show that the freshwater biodiversity crisis is intensifying as hydropower expansion, land‐use change, and climate warming act together to erode species and ecosystem functions (Reid et al. 2019; Grill et al. 2019).

The Lancang River Basin (LRB, upper Mekong) lies on the southeastern margin of the Tibetan Plateau and is a hotspot of cold‐water fish diversity and endemism in China. Over recent decades, cascaded hydropower dams and the spread of non‐native species have altered thermal and hydrological regimes and fragmented longitudinal connectivity (Ziv et al. 2012). Documented outcomes include spatial restructuring and declines in the taxonomic and phylogenetic diversity of fish assemblages (Zhang et al. 2018, 2019; Villéger et al. 2011). Loss of free‐flowing segments and barrier‐driven fragmentation increases extinction risk because dispersal pathways are severed across dendritic river networks (Grill et al. 2019; Fagan 2002).

**FIGURE 1:**
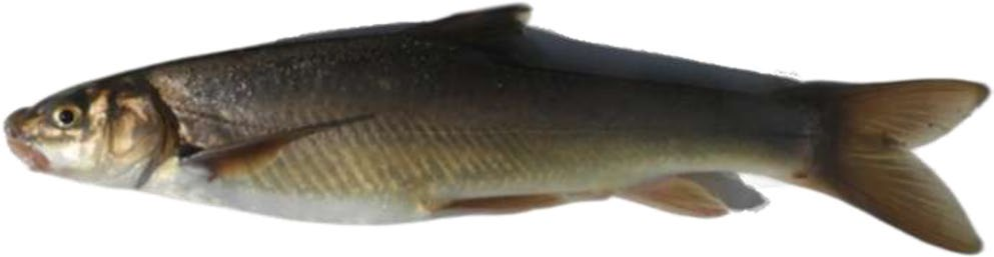
*Schizothorax lantsangensis* . (Image cited from: National Animal Specimen Resource Bank, http://museum.ioz.ac.cn/).

*Schizothorax lantsangensis* (Figure 1) is an endemic schizothoracine cyprinid restricted to the upper and middle LRB with ecological and regional fishery relevance. It is currently listed as Data Deficient on the IUCN Red List, which reflects limited population information despite clear exposure to basin‐scale pressures (Huckstorf 2013). Within the LRB, hydropower‐induced fragmentation, altered water temperature, and cumulative human disturbance have been linked to erosion of native fish diversity. These mechanisms support conservation concern for *S. lantsangensis* (Zhang et al. 2018, 2019; Villéger et al. 2011).

Climate change is expected to reorganize high‐elevation river habitats. Warming of river water, shifts in the magnitude and timing of runoff, and more frequent extremes are projected across the Tibetan Plateau, a region with strong climate sensitivity (Cui et al. 2021; Van Vliet et al. 2013; Wanders et al. 2019). Schizothoracids show high thermal sensitivity, and growth responses along elevation gradients indicate vulnerability to warming (Ding et al. 2016). High‐resolution global river‐temperature modeling now provides validated thermal constraints for habitat assessment, and evaluations of CMIP6 simulations over the Plateau support the use of scenario ensembles for ecological projections (Cui et al. 2021; Van Vliet et al. 2013; Wanders et al. 2019; O'Neill et al. 2016).

Studies from neighboring Plateau basins project elevational shifts and, in many cases, contractions of suitable habitats for cold‐water endemics. They also highlight the barrier effects of hydropower on connectivity (Mu et al. 2022). However, there is still no basin‐wide assessment of *S. lantsangensis* that works at the river‐network scale and jointly considers runoff, seasonal water temperature, and topographic and bioclimatic gradients.

To address this gap, we use a presence‐only species distribution modeling framework (MaxEnt) to quantify the relative importance of environmental predictors, map current potential suitability, and project mid‐ and late‐century changes under SSP126, SSP245, SSP370, and SSP585 (Phillips et al. 2006; Elith et al. 2011; O'Neill et al. 2016).

This study aims to (i) delineate the current spatial distribution of *Schizothorax lantsangensis* and identify the key environmental drivers; (ii) project mid‐ and late‐century changes in habitat suitability under CMIP6 scenarios (SSP126, SSP245, SSP370, SSP585); and (iii) characterize ecological response patterns to river runoff, seasonal water temperatures, and elevation using an optimized MaxEnt framework.

Grounded in the above rationale, we hypothesize that (1) suitable habitats are presently concentrated in the upper–middle reaches of the Lancang River Basin; (2) future warming will generally contract and fragment suitable habitats, with stronger effects under higher forcing and in the 2090s; and (3) the probability of occurrence exhibits distinct non‐linear thresholds to runoff, seasonal water temperatures, and elevation, beyond which suitability declines sharply.

## 2 Materials and Methods

### 2.1 Overview of the Study Area

The LRB, situated in southwestern China, spans longitudinally from 94° E to 102° E and latitudinally from 21° N to 34° N (Wang et al. 2025). The average temperature varies significantly across different sections of the basin: the upper reaches have average annual temperatures ranging from −3°C to 3°C, the middle reaches from 12°C to 15°C, and the lower reaches from 15°C to 18°C. Precipitation also shows spatial variability; the upper reaches receive 900–1100 mm of annual precipitation, while the middle and lower reaches, characterized by a northern subtropical climate, receive 1000–1600 mm annually (Li et al. 2002; Wang et al. 2024). The mean elevation of the LRB is approximately 3058 m. The upper reaches lie above 4000 m and are relatively flat, except for areas with high, rugged snow‐covered peaks. The middle reaches are defined by steep valleys and a pronounced riverbed gradient, with elevations ranging from 1200 to 5000 m. In contrast, the lower reaches are relatively flat, with an average elevation of around 1200 m (Dou et al. 2025).

### 2.2 Environmental Factor Data Preparation and Processing

#### 2.2.1 Acquisition of Environmental Factor Data

The 19 climate variables (Table S1) used in this study were downloaded from the WorldClim database (http://www.world‐clim.org, Version 2.1, 1 km spatial resolution) on 14 November 2024. Three topographic variables (elevation, slope, aspect) were obtained from the Computer Network Information Center of the Chinese Academy of Sciences and the International Science Data Platform (http://www.gscloud.cn, 30 m spatial resolution) on the same date. Watershed river flow accumulation data came from the Earth Environment Data Sharing Platform (http://www.earthenv.org, 1 km spatial resolution) and were accessed on 17 November 2024. Water temperature data were acquired from the Zenodo platform (https://doi.org/10.5281/zenodo.3337659) on 20 November 2024; for this dataset, monthly water temperatures of the Lancang River Basin (LRB) in typical wet (2001) and dry years (2008) were extracted first, and ArcGIS was then used to calculate the average water temperatures for spring, summer, autumn, and winter in both year types. This water temperature dataset has a spatial resolution of 10 km, and the dynamic water temperature model (DynWat) it adopts has been validated, showing a root mean square error of 0.2°C and a bias of 0.7°C across 358 global sites (Wanders et al. 2019).

Following previous studies on other fish species (Schaefer and Arroyave 2010; Xia et al. 2024; Zheng et al. 2024; Frederico et al. 2014), this study selected the above‐mentioned climate, topographic and river flow accumulation data. The selection of average seasonal water temperature data stratified by typical wet and dry years was tailored to *Schizothorax lantsangensis* : as a cold‐water species endemic to the LRB, *S. lantsangensis* depends on stable thermal conditions for core life‐history processes, including reproduction, foraging, and overwintering, all of which are highly sensitive to water temperature changes (Mu et al. 2022) (Shen et al. 2015). Meanwhile, the LRB has significant differences in runoff between wet and dry years (Wang et al. 2024; Dou et al. 2025), which directly affect water temperature stability. Covering both wet and dry year types ensures the study captures the actual thermal conditions the species encounters in its natural habitat.

Acquisition of Future Environmental Data: Future climate data were obtained from the WorldClim database based on the EC‐Earth3‐Veg climate model under the sixth phase of the Coupled Model Intercomparison Project (CMIP6) for the Shared Socioeconomic Pathways (SSP)126, SSP245, SSP370, and SSP585 scenarios (Table 1). This model has been verified as highly suitable for simulating the climate of the southern Tibetan Plateau (Zhu and Yang 2020). Data were obtained for the 2070s (2061–2080) and 2090s (2081–2100) periods with a spatial resolution of 1 km. The SSP126 scenario is a low‐emission pathway aiming for net‐zero emissions by 2050, with global temperature rise limited to 2°C by 2100; SSP245 represents moderate emissions with a 3°C rise by 2100; SSP370 assumes high emissions, resulting in a 4.1°C temperature rise by 2100; and SSP585 represents the highest emissions, with a projected 5°C rise by 2100 (Riahi et al. 2017).

**TABLE 1:** Four emission scenarios under the EC‐Earth3‐veg model.

Scenario name	Radiative forcing level	Scenario description
SSP126	Low forcing scenario	Global CO_2_ emissions are significantly reduced, reaching net‐zero around 2050. By 2100, the average global temperature increase is projected to be approximately 2°C.
SSP245	Moderate forcing scenario	CO_2_ emissions remain near current levels until mid‐century, then begin to decline but do not reach net‐zero by 2100. The average global temperature increase is projected to be around 3°C.
SSP370	Medium‐to‐high forcing scenario	CO_2_ emissions continue to rise throughout the century. By 2100, the average global temperature is expected to increase by approximately 4°C.
SSP585	High forcing scenario	CO_2_ emissions roughly double by 2050 under current trends. By 2100, the average global temperature increase is projected to reach approximately 5°C (Riahi et al. 2017)

#### 2.2.2 Environmental Variable Correlation Analysis and Selection

Given the significant correlations among the 19 climate variables (Table S1), Spearman's rank correlation analysis was conducted using ENMTools (Warren et al. 2010, 2021) software (Figure S1). ENMTools is a tool used for analyzing ecological niche models to address basic evolutionary ecological and biogeographical questions. The software was used to generate a correlation heatmap of the 19 climate variables in R version 4.4.2. If any two ecological factors exhibited a correlation coefficient |r| ≥ 0.8, only the climate variables that were most ecologically significant for the survival of *Schizothorax lantsangensis* were retained. The final selection of six climate variables for model construction included Bio2 (mean diurnal temperature range), Bio3 (isothermality), Bio14 (precipitation of the wettest month), Bio15 (precipitation seasonality), Bio17 (precipitation of the driest quarter), and Bio19 (precipitation of the coldest quarter). Bio2 and Bio3 influence temperature stability, which is critical for the growth and survival of *Schizothorax lantsangensis* , as the species is sensitive to temperature fluctuations. Bio14 and Bio15 control water availability and flow consistency, factors that are important for spawning and the persistence of suitable habitats. Bio17 affects water availability during dry periods, which can lead to reduced habitat connectivity, particularly during low‐flow seasons. Bio19 is crucial for maintaining cold‐water refugia, especially during winter, ensuring that the species has access to suitable thermal conditions for overwintering. These, together with the hydrological and topographic variables, were used to construct the MaxEnt model (Table 2). All environmental variables were resampled to a consistent spatial resolution of 1 km using ArcGIS software.

**TABLE 2:** List of environmental variables used for modeling the distribution of *S. lantsangensis* .

Variable	unit	Data sources	Scale
Mean Diurnal Range (Bio2)	°C	http://www.world‐clim.org	1 km
Isothermality (Bio3)	/	http://www.world‐clim.org	1 km
Precipitation of Driest Month (Bio14)	mm	http://www.world‐clim.org	1 km
Precipitation Seasonality (Bio15)	/	http://www.world‐clim.org	1 km
Precipitation of Driest Quarter (Bio17)	mm	http://www.world‐clim.org	1 km
Precipitation of Coldest Quarter (Bio19)	mm	http://www.world‐clim.org	1 km
Flow accumulation	/	http://www.earthenv.org	1 km
Elevation	m	http://www.gscloud.cn	30 m
Slope	°	http://www.gscloud.cn	30 m
Aspect	°	http://www.gscloud.cn	30 m
The water temperature in spring of a typical wet year (WT‐Spring_TWY)	°C	https://doi.org/10.5281/zenodo.3337659	10 km
The water temperature in summer of a typical wet year (WT‐Summer_TWY)	°C	https://doi.org/10.5281/zenodo.3337659	10 km
The water temperature in autumn of a typical wet year (WT‐Autumn_TWY)	°C	https://doi.org/10.5281/zenodo.3337659	10 km
The water temperature in winter of a typical wet year (WT‐Winter_TWY)	°C	https://doi.org/10.5281/zenodo.3337659	10 km
The water temperature in spring of a typical dry year (WT‐Spring_TDY)	°C	https://doi.org/10.5281/zenodo.3337659	10 km
The water temperature in summer of a typical dry year (WT‐Summer_TDY)	°C	https://doi.org/10.5281/zenodo.3337659	10 km
The water temperature in autumn of a typical dry year (WT‐Autumn_TDY)	°C	https://doi.org/10.5281/zenodo.3337659	10 km
The water temperature in winter of a typical dry year (WT‐Winter_TDY)	°C	https://doi.org/10.5281/zenodo.3337659	10 km

### 2.3 Collection and Processing of Species Distribution Data

**FIGURE 2:**
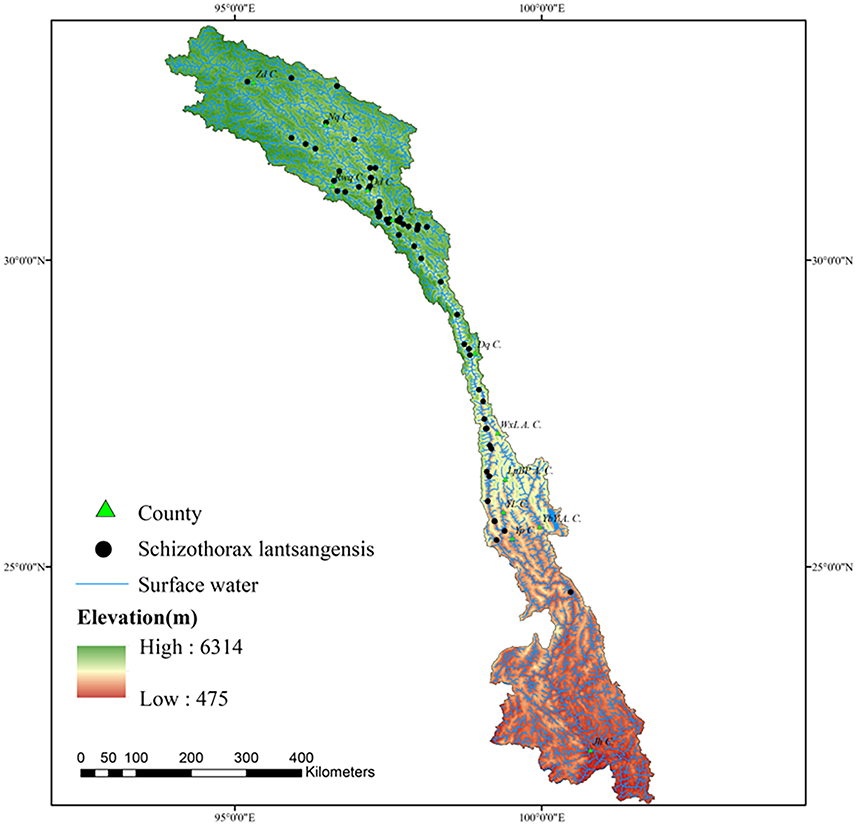
Research area and distribution of *Schizothorax lantsangensis* .

A total of 77 occurrence records for *Schizothorax lantsangensis* were compiled in this study, comprising 32 (Table S2) records extracted from published literature (Jin et al. 2022, 2024; Liu et al. 2016; Gao 2023; Chen 2017; Zhu et al. 2023; Wang 2019) and 45 additional points obtained through field surveys (Figure 2). To reduce the risk of model overfitting caused by spatial autocorrelation, redundant records located within the same grid cell were eliminated using the ENMTools toolkit (Warren et al. 2010, 2021). This R‐based toolkit (Warren et al. 2021) facilitates efficient spatial processing of species occurrence data, complementing its core function of ecological niche model comparison (Warren et al. 2010).

### 2.4 MaxEnt Model Configuration and Accuracy Evaluation

#### 2.4.1 Model Parameter Settings

The Percent Contribution (PC) and Permutation Importance (PI) are two standard metrics employed in the MaxEnt model to assess the influence of environmental variables on species distribution. PC represents the percentage contribution of each variable during model training, with higher values indicating greater relative importance. PI measures the drop in model performance (AUC) when each variable is randomly permuted, where a greater reduction indicates higher importance (Yao et al. 2023). Additionally, the Jackknife method is frequently applied to evaluate the relative importance of each variable (Bradley et al. 2010). This method includes three procedures: (1) model training with all variables except one (represented as “without variable”), (2) training with each variable alone (represented as “with only variable”), and (3) training with all variables. Comparative analysis of these results enables the identification of variables with strong predictive power (Figure 5) (Peterson and Cohoon 1999).

Model performance in MaxEnt is also strongly influenced by the selection of Feature Combinations (FCs) and Regularization Multipliers (RMs). MaxEnt provides five types of FCs: linear, quadratic, product, hinge, and threshold (Merow et al. 2013). The linear feature captures direct linear relationships between environmental variables and species presence, while the quadratic feature represents parabolic associations. Product features model interactions between variables; hinge features reflect piecewise, nonlinear ecological responses; and threshold features simulate abrupt ecological shifts when variables surpass critical thresholds. Combinations of these five feature types can generate up to 31 distinct model configurations, enabling flexible adaptation to complex ecological patterns.

The RM is a critical parameter in MaxEnt that controls model complexity by smoothing response curves, thereby mitigating the risk of overfitting (Elith et al. 2011). In this study, 40 RM values ranging from 0.1 to 4.0 (in increments of 0.1) were systematically tested. Model calibration and optimization were conducted using the kuenm package in R (Cobos et al. 2019), which applies a suite of evaluation metrics—including the Akaike Information Criterion corrected for small sample sizes (AICc), omission rates, and the Area Under the Curve (AUC)—to ensure model robustness and reproducibility. Specifically, the kuenm package is specialized for the detailed development of MaxEnt‐based ecological niche models (ENMs), enabling automated screening of optimal feature combinations (FCs) and regularization multipliers (RMs), quantitative comparison of model performance via multi‐metric evaluation, and effective mitigation of overfitting risks, which are key to the accuracy of our habitat suitability predictions (Cobos et al. 2019). Species occurrence records were randomly partitioned into training (75%) and testing (25%) subsets, and each model was replicated ten times. Jackknife analysis was used to evaluate the relative importance of the selected environmental variables. All other model parameters were kept at their default settings.

#### 2.4.2 Model Accuracy Evaluation

Model performance was assessed using the Receiver Operating Characteristic (ROC) curve and the corresponding area under the curve (AUC) value, both of which are widely accepted as reliable indicators of predictive accuracy in species distribution modeling (Wang et al. 2007). The ROC curve characterizes the trade‐off between sensitivity (true positive rate) and 1‐specificity (false positive rate), whereas the AUC provides a single scalar value summarizing the model's ability to distinguish between suitable and unsuitable habitats across all threshold levels (Hanley and Mcneil 1982). In this study, AUC values derived from Jackknife resampling were used to evaluate the overall predictive performance of the model and to quantify the relative contributions of individual environmental variables.

Habitat suitability for *S. lantsangensis* was further reclassified using the maximum test sensitivity plus specificity (MTSPS) threshold (Fordjour et al. 2022). This threshold optimization method provides a balanced trade‐off between sensitivity—the model's ability to accurately identify suitable habitats—and specificity—the ability to correctly exclude unsuitable areas. The MTSPS criterion is particularly appropriate for presence‐only modeling approaches like MaxEnt, where absence data are unavailable and species occurrence records are often sparse (Liu et al. 2013).

## 3 Results and Analysis

### 3.1 Model Parameter Selection and Accuracy Evaluation

**FIGURE 3:**
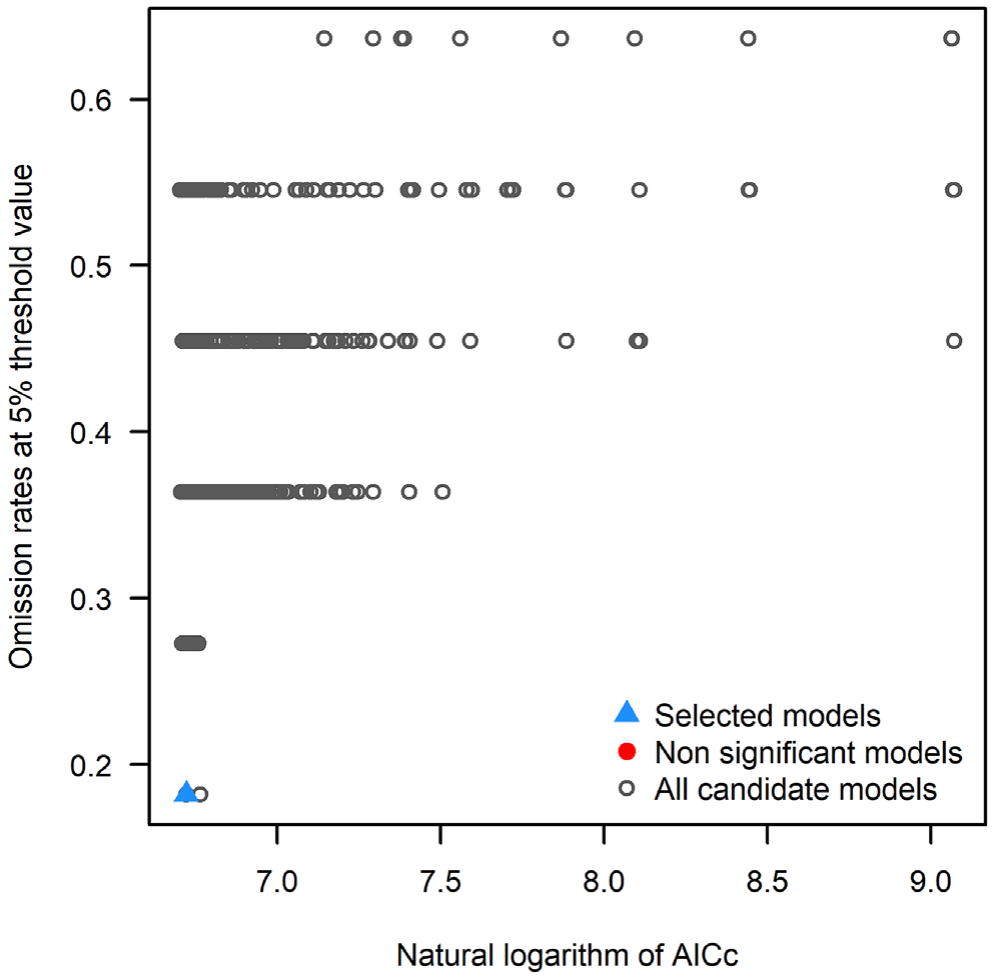
R program model selection results.

To optimize model performance, the MaxEnt model was calibrated using the kuenm package (Cobos et al. 2019) in R. Models with omission rates ≤ 10% and statistically significant performance were first selected. Among these, the configuration with the lowest delta AICc for small sample sizes (ΔAICc = 0) was identified as optimal (Warren and Seifert 2011). This final model adopted a regularization multiplier (RM) of 0.1 and a quadratic (Q) feature combination. The omission rate of the best‐performing model was 0.08 (Figure 3), indicating a low risk of overfitting.

**FIGURE 4:**
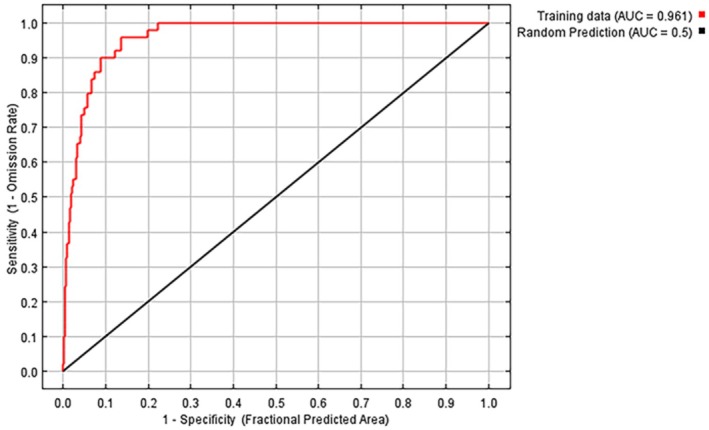
The receiver operating characteristic (ROC) curve in MaxEnt model.

The predictive accuracy of the MaxEnt model was evaluated using the Area Under the Curve (AUC) of the Receiver Operating Characteristic (ROC) curve. AUC values range from 0 to 1, where values of 0.5–0.6 indicate poor performance, 0.6–0.7 fair, 0.7–0.8 moderate, 0.8–0.9 good, and values above 0.9 are considered excellent (Phillips et al. 2006). In this study, the MaxEnt model achieved an average AUC of 0.961 across replicate runs, reflecting excellent predictive capacity and a high degree of model reliability (Figure 4).

### 3.2 Key Environmental Drivers of *S. lantsangensis* Distribution

**FIGURE 5:**
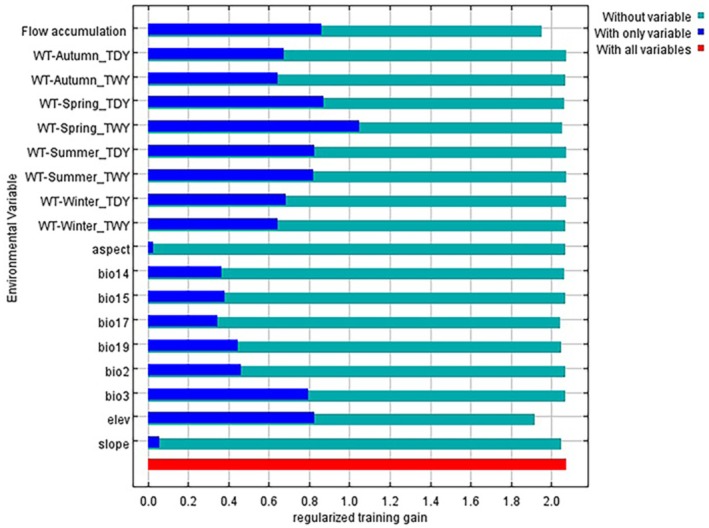
Importance of variables in training data for Jackknife test.

The importance of environmental variables was assessed using percent contribution and permutation importance metrics (Table 3). In addition, the Jackknife test was applied to evaluate the explanatory power of each individual variable (Figure 5). Among all predictors, flow accumulation showed the highest percent contribution (35.7%), followed by water temperature in winter of a typical dry year (28.0%). Although elevation (6.3%) and water temperature in spring of a typical dry year (3.0%) had lower percent contributions, their permutation importance values were notably high, at 30.0% and 43.3%, respectively. In contrast, the percent contribution and permutation importance of other environmental variables were relatively low. Overall, flow accumulation, water temperature in winter of a typical dry year, elevation in the LRB, and water temperature in spring of a typical dry year were identified as the key environmental factors influencing the distribution of *S. lantsangensis* .

**TABLE 3:** Permutation importance of variables affecting the distribution of *S. lantsangensis* .

Variable	Percent contribution	Permutation importance
Flow accumulation	35.7	3.7
WT‐Winter_TDY	28	3
WT‐Spring_TWY	14.4	0.3
elev	6.3	30
bio2	3.2	1.4
WT‐Winter_TWY	3.1	0
WT‐Spring_TDY	3	43.3
bio17	2.1	0.5
bio19	1.4	0.5
WT‐Autumn_TWY	0.8	15.3
slope	0.8	0
bio14	0.4	0.3
aspect	0.4	0.3
WT‐Summer_TDY	0.1	1.4
bio3	0	0
bio15	0	0.1
WT‐Summer_TWY	0	0
WT‐Autumn_TDY	0	0

### 3.3 Species Response to Key Environmental Variables

**FIGURE 6:**
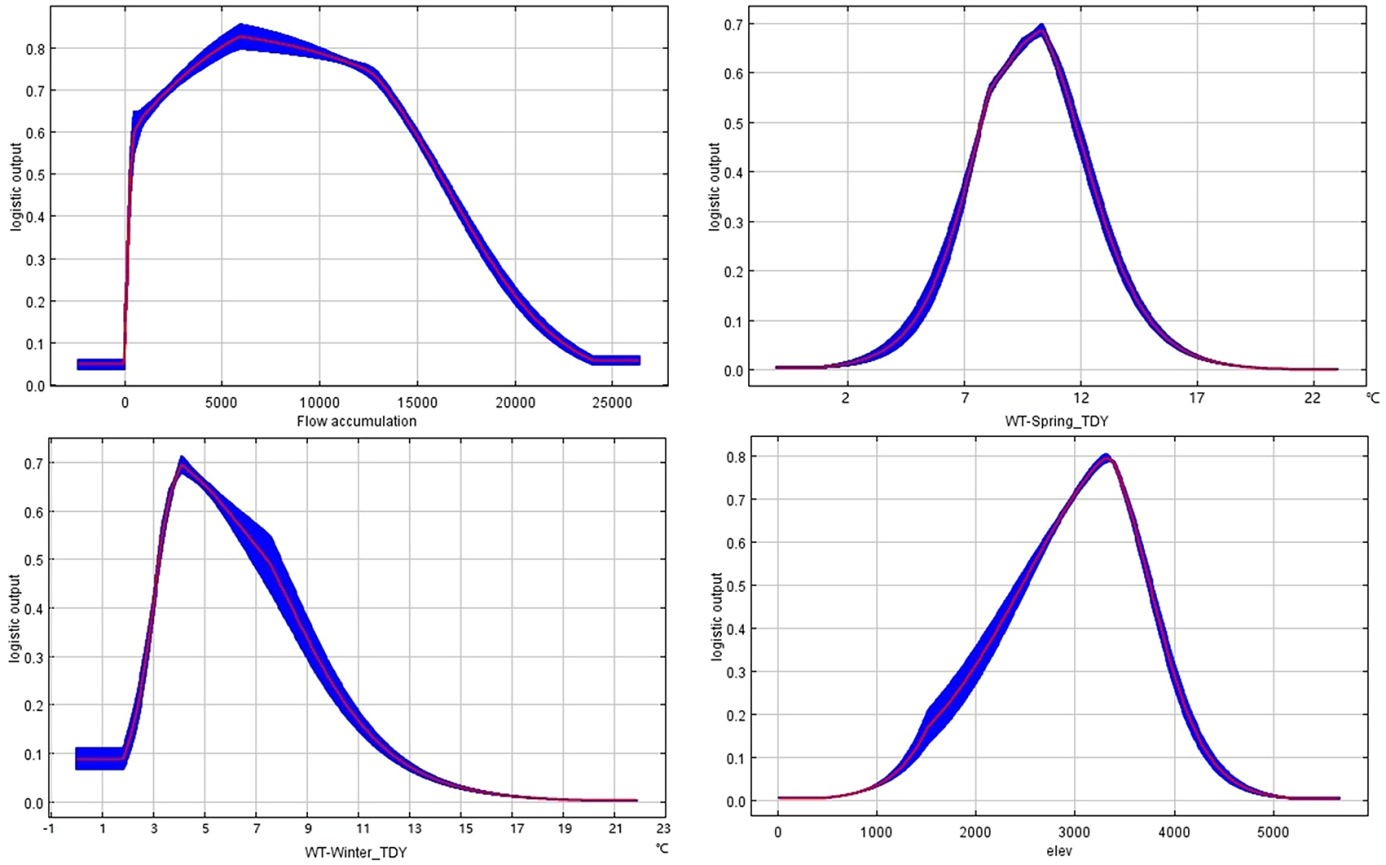
Response curve of key environmental variables:Flow accumulation, water temperature in spring of typical dry year (WT‐Spring_TDY), water temperature in winter of typical dry year (WT‐Winter_TDY), and elevation (elev).

The MaxEnt model produced response curves illustrating the probability of *S. lantsangensis* occurrence along gradients of key environmental variables (Figure 6). Based on the logistic output, a threshold of 0.6 was applied to delineate areas with high habitat suitability. The optimal environmental conditions associated with a high likelihood of species presence were as follows: flow accumulation between 855 and 14,835.5, water temperature in winter of a typical dry year between 3.5°C and 6.0°C, elevation between 2722 m and 3650 m, and water temperature in spring of a typical dry year between 8.5°C and 11.0°C. These response curves suggest that within the study area (the Lancang River Basin), *S. lantsangensis* prefers moderately high elevations, low winter temperatures, and areas with higher flow accumulation.

### 3.4 Current Habitat Suitability and Distribution of *S. lantsangensis*

To distinguish suitable from unsuitable habitats, a logistic threshold was applied based on the Maximum Test Sensitivity plus Specificity (MTSPS = 0.051), which provides an optimal balance between omission and commission errors. Grid cells with predicted occurrence probabilities exceeding this threshold were classified as suitable habitats (Fordjour et al. 2022).

**FIGURE 7:**
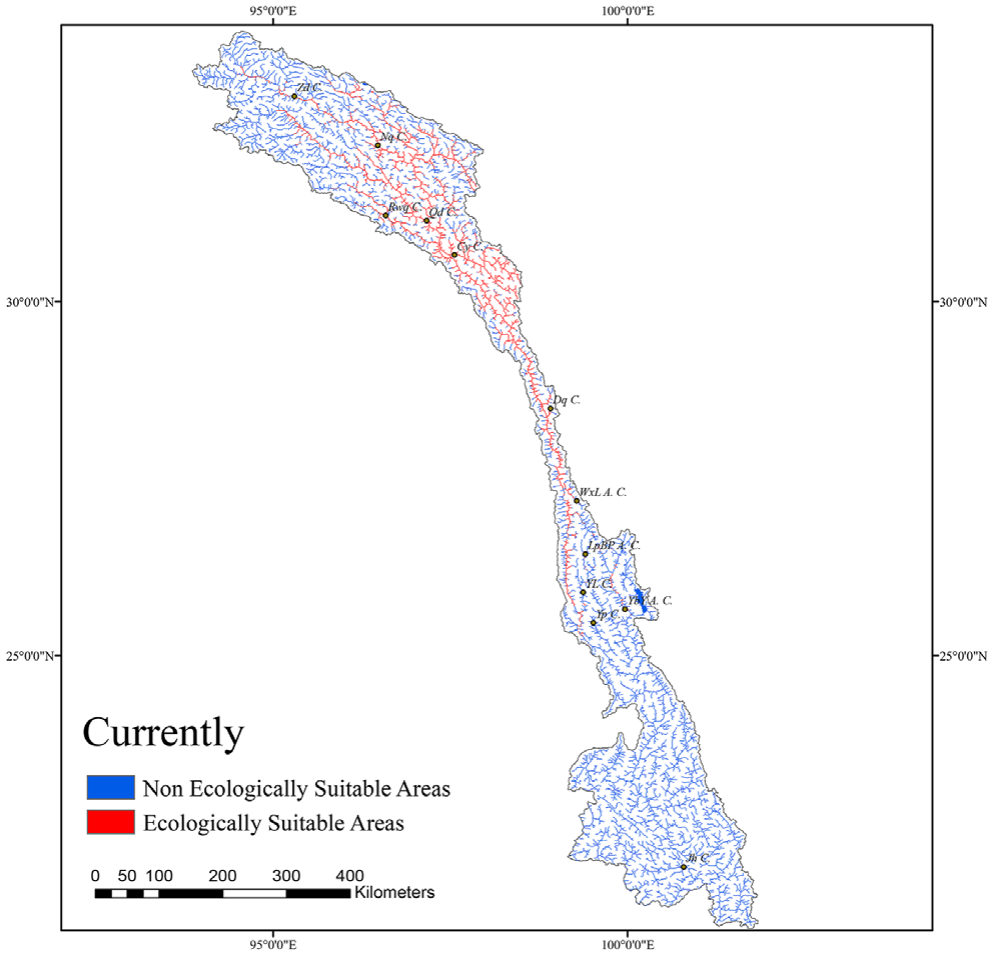
Distribution of suitable habitats for *S. lantsangensis* .

The binary suitability map generated by the MaxEnt model was imported into ArcGIS to visualize the current spatial distribution of *S. lantsangensis* (Figure 7). According to river length calculations, the total river network within the study area spans 21,609.17 km, of which 4776.67 km (22.1%) were identified as suitable habitat. These suitable sections are primarily concentrated in the upper and middle reaches of the LRB, especially in river segments near Nangqen County (NQ County), Qamdo City (QD City), Chagyab County (CY County), and Dêqên County (DQ County). All known species occurrence records fell within the predicted suitable areas, further supporting the robustness and spatial accuracy of the model.

### 3.5 Future Habitat Suitability Under Climate Change Scenarios

**FIGURE 8:**
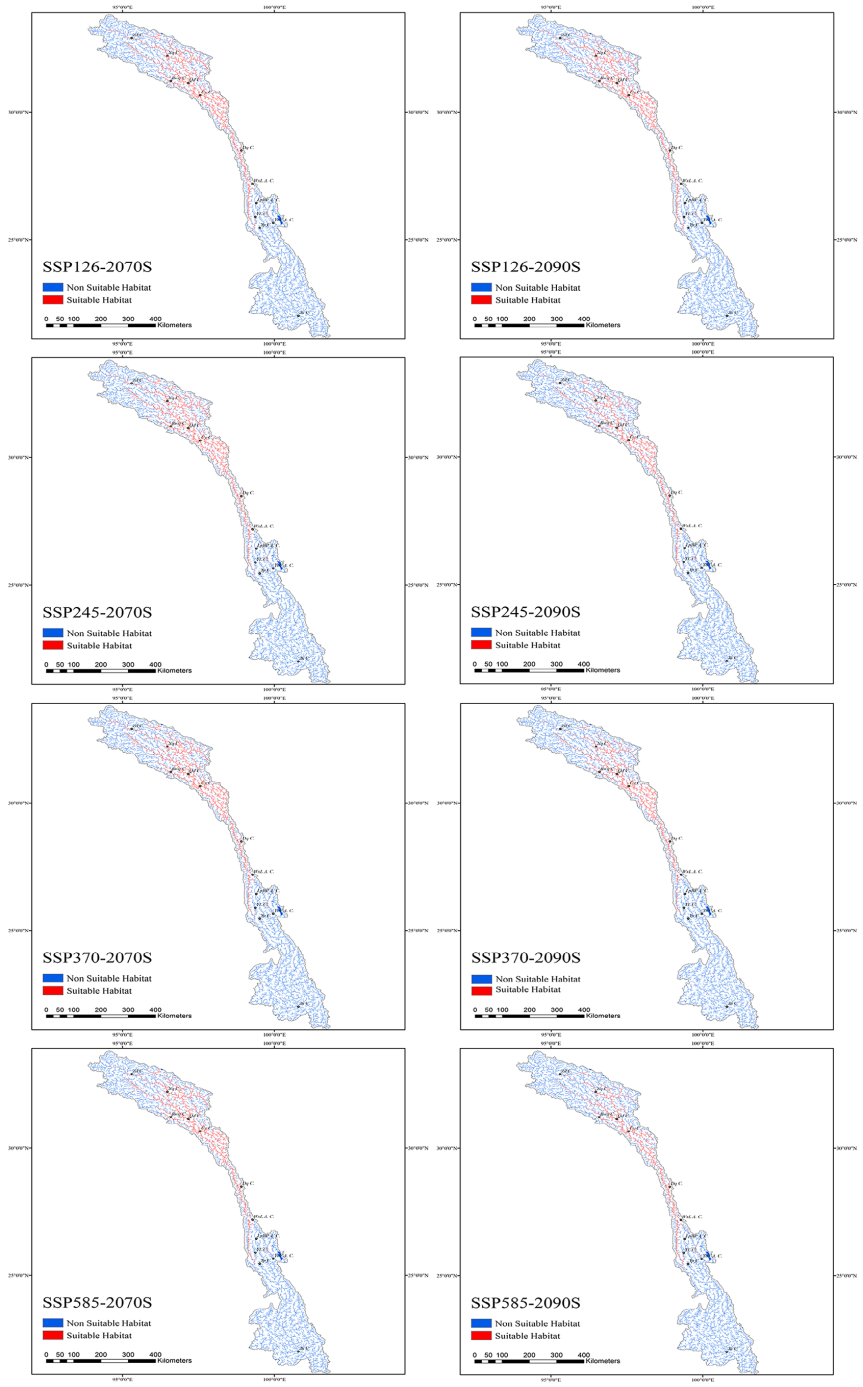
Suitable habitats for *S. lantsangensis* under future climate scenarios.

**FIGURE 9:**
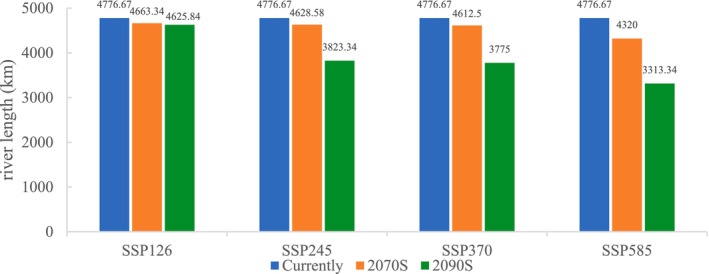
Suitable habitats river length for *S. lantsangensis* under future climate scenarios.

Using the current distribution of suitable habitat as a baseline, future projections under various climate change scenarios consistently indicate a contraction in the suitable habitat range of *S. lantsangensis* across all SSPs (Figures 8 and 9). The MaxEnt model predicts that suitable river habitat will gradually decline with increasing radiative forcing, and the extent of habitat loss will intensify over time. Under the low‐emission scenario SSP126, suitable habitat is projected to decrease slightly to 4663.34 km by the 2070s, representing a 2.37% reduction compared to the current extent. By the 2090s, it is expected to decline further to 4625.84 km (3.16% reduction). In the intermediate scenario SSP245, habitat length is projected to decline moderately to 4628.58 km in the 2070s (3.10% reduction), followed by a sharper decrease to 3823.34 km by the 2090s (19.96% loss). Under SSP370, which represents a medium‐to‐high emissions pathway, a similar pattern is observed, with habitat length declining to 4612.50 km in the 2070s (3.44% reduction) and to 3775.00 km in the 2090s (20.97% reduction). The most significant contraction occurs under the high‐emission scenario SSP585, where suitable habitat length is projected to fall to 4320.00 km in the 2070s (9.56% reduction) and further to 3313.34 km by the 2090s, amounting to a substantial 30.63% decline relative to the present. These projections underscore the strong negative influence of climate change on the ecological suitability of the LRB for *S. lantsangensis* , particularly under high‐emission trajectories. The anticipated loss of suitable habitat highlights the species' vulnerability to climate‐induced environmental changes and reinforces the urgency of implementing targeted conservation and adaptive management strategies.

## 4 Conclusion and Discussion

### 4.1 Discussion

#### 4.1.1 Model Performance Evaluation

The MaxEnt model exhibited strong predictive performance, as reflected by a high AUC value of 0.961, meeting the criterion for “excellent” model accuracy (Guo et al. 2020). To enhance robustness and reduce the risk of overfitting, the model was optimized using the kuenm package in R, which facilitated automated calibration of feature combinations (FCs) and regularization multipliers (RMs). The final model was selected based on the lowest corrected Akaike Information Criterion (AICc), a statistically significant omission rate, and optimal discriminatory power.

In addition, spatial autocorrelation among occurrence records—particularly due to clustering in certain river segments—was mitigated through spatial filtering in ENMTools. This process removed redundant records within identical grid cells, thereby reducing spatial bias and improving model generalizability. The integration of parameter optimization and spatial thinning contributed to the reliability and reproducibility of the predicted habitat suitability for *S. lantsangensis* .

#### 4.1.2 Key Environmental Drivers of Habitat Suitability

This study identified river flow, water temperature, and elevation as the primary natural environmental factors influencing the potential distribution of *S. lantsangensis* . These findings are consistent with previous research on plateau fish species (Mu et al. 2022; Liu et al. 2016; Soumyadip et al. 2021), where such variables have been shown to significantly affect growth, reproduction, and survival. The seasonal water temperature variables identified by the MaxEnt model correspond closely with key life‐history stages of *S. lantsangensis* . Spring temperature affects spawning and embryonic development, as spawning typically occurs from March to June when water temperatures range from 9°C to 15°C. The response curve shows optimal suitability at 8.5°C–11°C, consistent with this biological window. Summer temperature influences metabolic rate and growth, yet temperatures above approximately 25°C may cause feeding cessation and increased mortality. Autumn and winter temperatures regulate overwintering survival and energy storage, with model‐derived optimal ranges of 3.5°C–6.0°C, aligning with field observations. Environmental variables showed distinct roles in shaping the habitat suitability of *S. lantsangensis* . Although flow accumulation exhibited the highest percent contribution during model training, temperature variables—particularly WT‐Spring_TDY—showed the highest permutation importance and jackknife gain, indicating that water temperature is the primary determinant of habitat suitability, while flow accumulation mainly shapes the spatial distribution of suitable reaches by regulating river network connectivity, hydraulic conditions (e.g., water depth and flow velocity), and habitat continuity (Bai et al. 2025). This differentiation aligns with the ecological traits of *S. lantsangensis* as a cold‐water rheophilic fish, where temperature determines fundamental survival thresholds and hydrological processes dictate habitat spatial arrangement. Additionally, the probability of *S. lantsangensis* occurrence increases with elevation, peaking at approximately 3300 m before declining, a pattern that aligns with historical records of species presence and abundance (Zhu et al. 2023, 2022). This trend is likely influenced by associated environmental factors, such as flow velocity, dissolved oxygen levels, and substrate composition (Yang et al. 2024, 2025; Hansen et al. 2024), which vary with elevation and are critical to the species' habitat suitability.

#### 4.1.3 Current Distribution and Habitat Suitability Zoning

In current research, habitat suitability is commonly classified using natural breaks or fixed thresholds, typically dividing areas into four categories: highly suitable, moderately suitable, marginally suitable, and unsuitable. However, to more objectively and accurately delineate the potential habitat of *S. lantsangensis* , this study defined suitable areas as those with a Habitat Suitability Index (HSI) exceeding the Maximum Test Sensitivity plus Specificity (MTSPS) threshold (Liu et al. 2013).

According to the MaxEnt model results, the total length of suitable river habitat for *S. lantsangensis* was estimated at 4776.67 km, accounting for 22.1% of the LRB river network. These suitable areas are primarily concentrated in the upper and middle reaches of the LRB, particularly in river segments near NQ County, QD City, CY County, and DQ County. To promote sustainable development of *S. lantsangensis* populations, conservation efforts should prioritize these ecologically suitable zones. Human disturbances, including overfishing and the introduction of non‐native fish species, should be minimized. In parallel, stock enhancement programs should be implemented to restore and expand wild populations in key habitats.

#### 4.1.4 Changes in Suitable Habitat Under Future Climate Scenarios

A growing body of evidence suggests that climate change poses a substantial threat to biodiversity within the LRB (Gui et al. 2021; Dou et al. 2019). Compared to terrestrial ecosystems, freshwater ecosystems are generally more sensitive to climate fluctuations (Morrongiello et al. 2011), due to three core vulnerabilities: (1) Hydrological dependence: Stable water availability is critical, and climate‐driven changes in precipitation/runoff rapidly disrupt habitat integrity (Ratcliffe et al. 2025); (2) Restricted dispersal: Freshwater organisms are confined by river/lake boundaries, limiting their ability to escape unsuitable conditions (Dudgeon et al. 2006); (3) Narrow abiotic tolerance: Freshwater biota have stricter requirements for temperature/flow, with slight shifts exceeding survival thresholds (Morrongiello et al. 2011). The findings of this study indicate that under future warming scenarios, the ecological suitability of habitats for *S. lantsangensis* is projected to decline consistently. As a cold‐water fish inhabiting mountainous regions, *S. lantsangensis* is particularly sensitive to temperature variations, with water temperature identified as the dominant environmental driver influencing its distribution (Liu et al. 2016). Climate projections further predict increased temperature variability, along with a greater frequency and intensity of extreme weather events in the future (Thirumalai et al. 2017). These climatic shifts may lead to extended dry periods in low‐ and mid‐latitude regions, thereby reducing the availability of suitable river habitat for *S. lantsangensis* . Moreover, the LRB region, as a typical alpine transboundary river basin on the Tibetan Plateau, shares core ecological and hydrological vulnerabilities with similar regions globally—including high sensitivity of endemic fish habitats to climate change, tight coupling between abiotic‐biotic processes and climatic variations, and fragile ecosystem stability under cryosphere loss (Mu et al. 2022; Morovati et al. 2024; Anslan et al. 2020; Liu et al. 2024). With continued rises in carbon emissions and associated warming, the persistence of *S. lantsangensis* in its native range will face escalating ecological challenges.

It should be noted that flow accumulation was treated as a static topographic variable derived from the DEM and was not projected under future climate scenarios. Consequently, the predicted changes in habitat suitability primarily represent the effects of climate‐induced thermal shifts rather than potential alterations in discharge or hydrological connectivity. This simplification is reasonable for assessing temperature sensitivity but may underestimate the compound impacts of hydrological and thermal changes. Future studies should incorporate dynamic hydrological simulations or coupled water–temperature models to capture the interactive effects of changing flow regimes, runoff, and water temperature on habitat availability.

### 4.2 Conclusion

Using an optimized MaxEnt configuration (AUC = 0.961), we show that the distribution of *Schizothorax lantsangensis* is governed primarily by flow accumulation, winter (dry‐year) water temperature, elevation, and spring (dry‐year) water temperature. At present, 4776.67 km of river length (22.1% of the network) is suitable, concentrated in the upper–middle LRB near Nangqen (NQ), Qamdo (QD), Chagyab (CY), and Dêqên (DQ). Future warming is projected to reduce suitable habitat across scenarios, with losses reaching up to 30.63% by the 2090s under SSP585, underscoring urgent management needs.

Management implications based on these findings:

1. Priority protection and zoning. Designate and connect reserves in the identified county reaches and cold‐water tributaries within 2722–3650 m elevation to secure core populations and maintain dispersal.
2. Thermal‐refugia protection. Limit riparian deforestation and thermal pollution; where feasible, favor operations that reduce warm‐season heating in priority reaches, to keep spring reach temperatures near 8.5°C–11.0°C and winter near 3.5°C–6.0°C.
3. Environmental‐flow guidance. During March–June (spawning window), implement seasonal environmental‐flow releases that avoid extreme low flows in dry years and support hydraulics consistent with the high‐suitability range identified along high‐accumulation corridors.
4. Adaptive reinforcement with safeguards. Continue refining artificial breeding for targeted stock enhancement in priority reaches while managing genetic risks.
5. Monitoring benchmarks and climate screening. Use the quantified baseline (4776.67 km) and thresholds above as indicators for long‐term monitoring and as criteria to screen the siting and operations of infrastructure to minimize losses of future suitable habitat.

Contribution of this study. Beyond mapping suitability, we provide basin‐wide, spatially explicit priorities and operational thresholds (thermal, elevational, and hydrological) that managers can apply directly to reserve design, flow operations, and habitat restoration, while quantifying the magnitude and timing of climate‐driven losses to plan adaptive actions.

## Author Contributions

**Yuchen Xu:** conceptualization (equal), data curation (equal), formal analysis (equal), investigation (equal), validation (equal), writing – original draft (equal). **Jia Li:** funding acquisition (equal), investigation (equal), project administration (equal), resources (equal), supervision (equal), visualization (equal), writing – review and editing (equal). **Li Chang:** data curation (equal), funding acquisition (equal), investigation (equal), resources (equal), visualization (equal). **Ruidong An:** funding acquisition (equal), resources (equal), supervision (equal), visualization (equal), writing – review and editing (equal).

## Funding

This work was supported by the National Key Research and Development Program of China (2022YFC3204202), Guizhou Provincial High‐level Innovative Talents (GCC [2023] 103).

## Conflicts of Interest

The authors declare no conflicts of interest.

## Supporting information


**Table S1:** Bioclimatic variables.
**Table S2:** The literature records the distribution information of *S. lantsangensis* .
**Figure S1:** Analysis of correlation between climatic factors.

## Data Availability

The data supporting the findings of this study were obtained from the following databases, with detailed access dates provided: WorldClim database (https://worldclim.org/), accessed on 14 November 2024; Geospatial Data Cloud (https://www.gscloud.cn/home), accessed on 14 November 2024; EarthEnv database (http://www.earthenv.org), accessed on 17 November 2024; Zenodo platform (https://doi.org/10.5281/zenodo.3337659), accessed on 20 November 2024. All data sources complied with legal requirements. The codes and software used in this study are publicly available as follows: ENMTools: Used for environmental variable correlation analysis and spatial filtering of species occurrence records. Available on the Comprehensive R Archive Network (CRAN, https://cran.r‐project.org/web/packages/ENMTools/) and GitHub (https://github.com/danlwarren/ENMTools) (Warren et al. 2010, 2021); kuenm package: Used for MaxEnt model calibration and optimization. Available on CRAN (https://cran.rproject.org/web/packages/kuenm/) and GitHub (https://github.com/marlonecobos/kuenm) (Cobos et al. 2019); MaxEnt: Used for species distribution modeling. Official version available at https://biodiversityinformatics.amnh.org/open_source/maxent/ (Elith et al. 2011; Warren and Seifert 2011). ArcGIS: Used for spatial data processing and visualization. Developed by Esri, available at https://www.esri.com/en‐us/arcgis/products/arcgis‐desktop/overview. The authors confirm that the data, codes, and software supporting the findings of this study are available within the article and its Supporting Information S1.
