# Supplementary material for: Climate‐Induced Changes in Habitat Suitability for a Cold‐Water Endemic Species in the Lancang River Basin: A Case Study of Schizothorax lantsangensis
Source: Ecol Evol. 2025 Dec 22;15(12):e72753. doi: 10.1002/ece3.72753 (PMC12719919; doi:10.1002/ece3.72753)
Supplement: Supplementary file 1 — Table S1: Bioclimatic variables. Table S2: The literature records the distribution information of S. lantsangensis . Figure S1: Analysis of correlation between climatic factors. [file ECE3-15-e72753-s001.docx]

Supporting information

**Climate-Induced Changes in Habitat Suitability for a Cold-Water Endemic Species in the LCJ Basin: A Case Study of Schizothorax lantsangensis**

Yuchen Xu^1^, Jia Li^1^, Li Chang^2*^, Ruidong An^1*^

^1^Sichuan Univ, Coll Water Resource & Hydropower, State Key Lab Hydraul & Mt River Engn, Chengdu, Peoples R China

^2^Guiyang Engineering Co., Ltd., Power China, Guiyang, Peoples R China

*Corresponding author

changl_gyy@powerchina.cn(Li Chang); anruidong@scu.edu.cn(Ruidong An)

Table S1 Bioclimatic variables.

| **variables** | Description | **variables** | Description | **variables** | | Description |
| --- | --- | --- | --- | --- | --- | --- |
| Bio1 | Annual mean temperature | Bio8 | Mean temperature of wettest quarter | Bio15 | | Precipitation seasonality |
| Bio2 | Mean monthly temperature range | Bio9 | Mean temperature of driest quarter | Bio16 | | Precipitation of wettest quarter |
| Bio3 | Isothermality | Bio10 | Mean temperature of warmest quarter | Bio17 | | Precipitation of driest quarter |
| Bio4 | Temperature seasonality | Bio11 | Mean temperature of coldest quarter | Bio18 | | Precipitation of warmest quarter |
| Bio5 | Max temperature of warmest month | Bio12 | Annual precipitation | Bio19 | | Precipitation of coldest quarter |
| Bio6 | Min temperature of coldest month | Bio13 | Precipitation of wettest month |  |  | |
| Bio7 | Temperature annual range | Bio14 | Precipitation of driest month |  |  | |

Table S2 The literature records the distribution information of ***S. lantsangensis***.

| Sampling site | longitude | latitude | Historical distribution point References |
| --- | --- | --- | --- |
| Miaowei-Gongguoqiao | 99.236°E | 25.749°N | Jin et al. 2022^[32]^ |
| Huangdeng-Dahuaqiao | 99.142°E | 26.482°N |  |
| Lidi Reservoir | 99.091°E | 27.257°N |  |
| Wunonglong | 98.981°E | 27.888°N |  |
| Biluo River | 99.062°E | 27.411°N | Chen et al. 2017^[35]^ |
| Biyu River | 99.153°E | 26.980°N |  |
| Deqing River | 99.176°E | 26.928°N |  |
| Yingpan | 99.145°E | 26.480°N |  |
| Quzika | 98.618°E | 29.112°N | Zhu et al. 2023^[36]^ |
| Rumei | 98.350°E | 29.643°N |  |
| Karuo | 97.350°E | 30.881°N |  |
| Zaqu | 97.209°E | 31.346°N |  |
| Jinhe | 97.332°E | 30.777°N |  |
| Requ | 97.287°E | 31.501°N |  |
| Wunonglong | 98.981°E | 27.888°N | Gao et al. 2023^[34]^ |
| Lidi | 99.047°E | 27.700°N |  |
| Rumei | 95.917°E | 31.996°N | Liu et al.2016^[33]^ |
| Maiqu | 97.468°E | 30.661°N |  |
| Seqqu | 96.610°E | 31.299°N |  |
| Liutong Jiang | 98.809°E | 28.556°N |  |
| Manwan | 100.471°E | 24.592°N |  |
| Biaocun | 99.120°E | 26.069°N |  |
| Jiuzhou | 99.233°E | 25.747°N |  |
| Wayao | 99.259°E | 25.441°N |  |
| Wunonglong | 99.142°E | 26.482°N | Jin et al. 2024^[37]^ |
| Lidi | 99.101°E | 26.554°N |  |
| Baijixun | 99.101°E | 27.258°N |  |
| Zhongpai | 98.981°E | 27.888°N |  |
| Lagu | 99.141°E | 25.257°N |  |
| Lamadeng | 99.236°E | 25.749°N |  |
| Gongguoqiao | 99.397°E | 25.591°N |  |
| Nangqian | 96.153°E | 31.894°N | Wang et al. 2019^[38]^ |


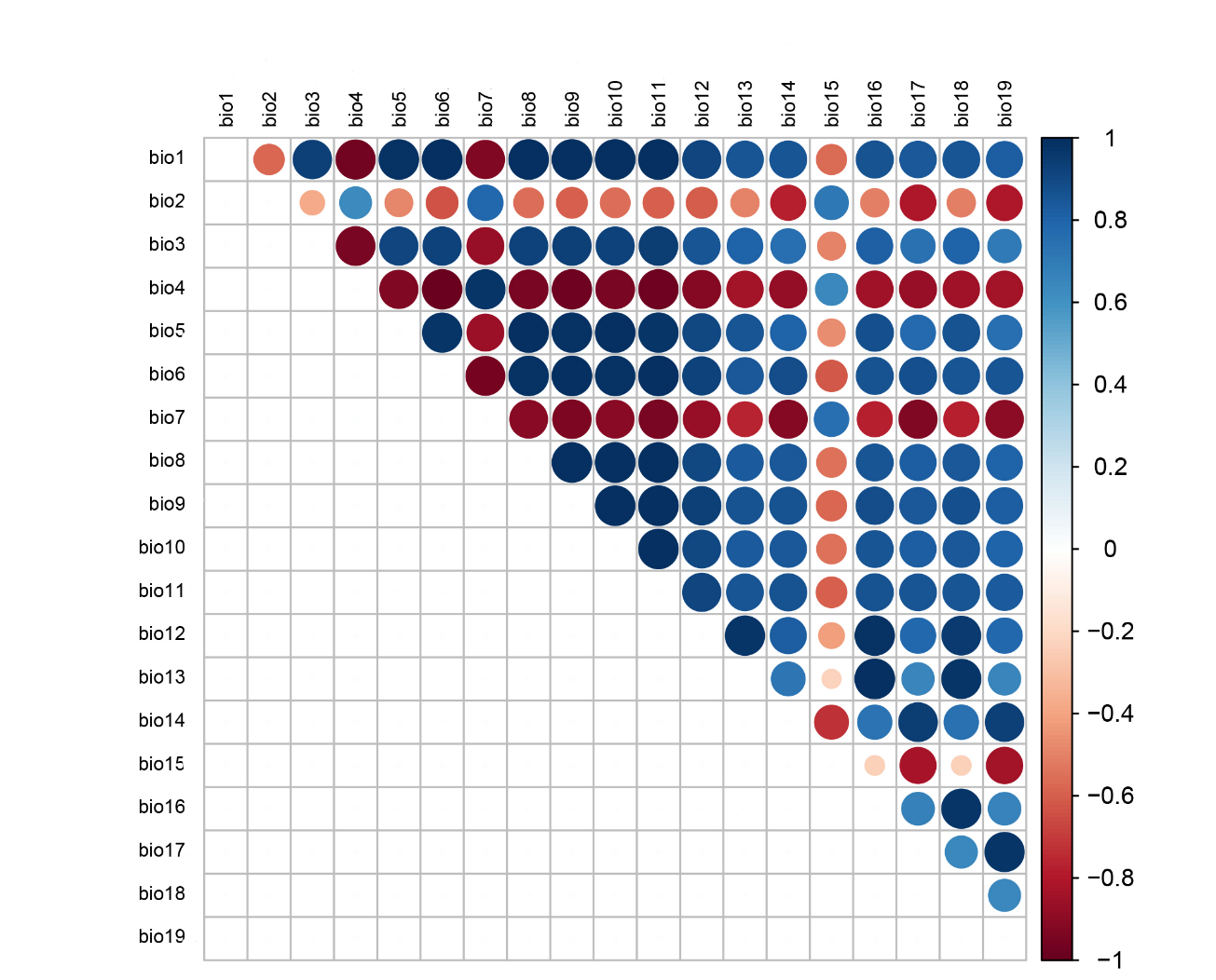


Figure S1 Analysis of correlation between climatic factors.
